# Supplementary material for: Synthesis, Characterization, Cytotoxicity Analysis and Evaluation of Novel Heterocyclic Derivatives of Benzamidine against Periodontal Disease Triggering Bacteria
Source: Antibiotics (Basel). 2023 Feb 2;12(2):306. doi: 10.3390/antibiotics12020306 (PMC9952644; doi:10.3390/antibiotics12020306)
Supplement: Supplementary file 1 [file antibiotics-12-00306-s001.zip › antibiotics-2188660-supplementary.pdf]

Supplementary Material

Synthesis, Characterization, Cytotoxicity Analysis and Evaluation of Novel Heterocyclic Derivatives of Benzamidine against Periodontal Disease Triggering Bacteria

Ramasamy Kavitha [1], Mohammad Auwal Sa'ad [1,2], Shivkanya Fuloria [3], Neeraj Kumar Fuloria [3,4,*], Manickam Ravichandran [1,2,5,*] and Pattabhiraman Lalitha [6]

[1] Department of Biotechnology, Faculty of Applied Science, AIMST University, Bedong 08100, Kedah, Malaysia
[2] Centre of Excellence for Vaccine Development (CoEVD), Faculty of Applied Science, AIMST University, Bedong 08100, Kedah, Malaysia
[3] Centre of Excellence for Biomaterials Engineering, Faculty of Pharmacy, AIMST University, Bedong 08100, Kedah, Malaysia
[4] Center for Transdisciplinary Research, Department of Pharmacology, Saveetha Institute of Medical and Technical Sciences, Saveetha Dental College and Hospital, Saveetha University, Chennai 600077, Tamil Nadu, India
[5] Mygenome, ALPS Global Holding, Kuala Lumpur 50400, Malaysia
[6] Department of Biochemistry, Faculty of Medicine, AIMST University, Bedong 08100, Kedah, Malaysia
* Correspondence: neerajkumar@aimst.edu.my (N.K.F.); ravichandran@aimst.edu.my (M.R.)

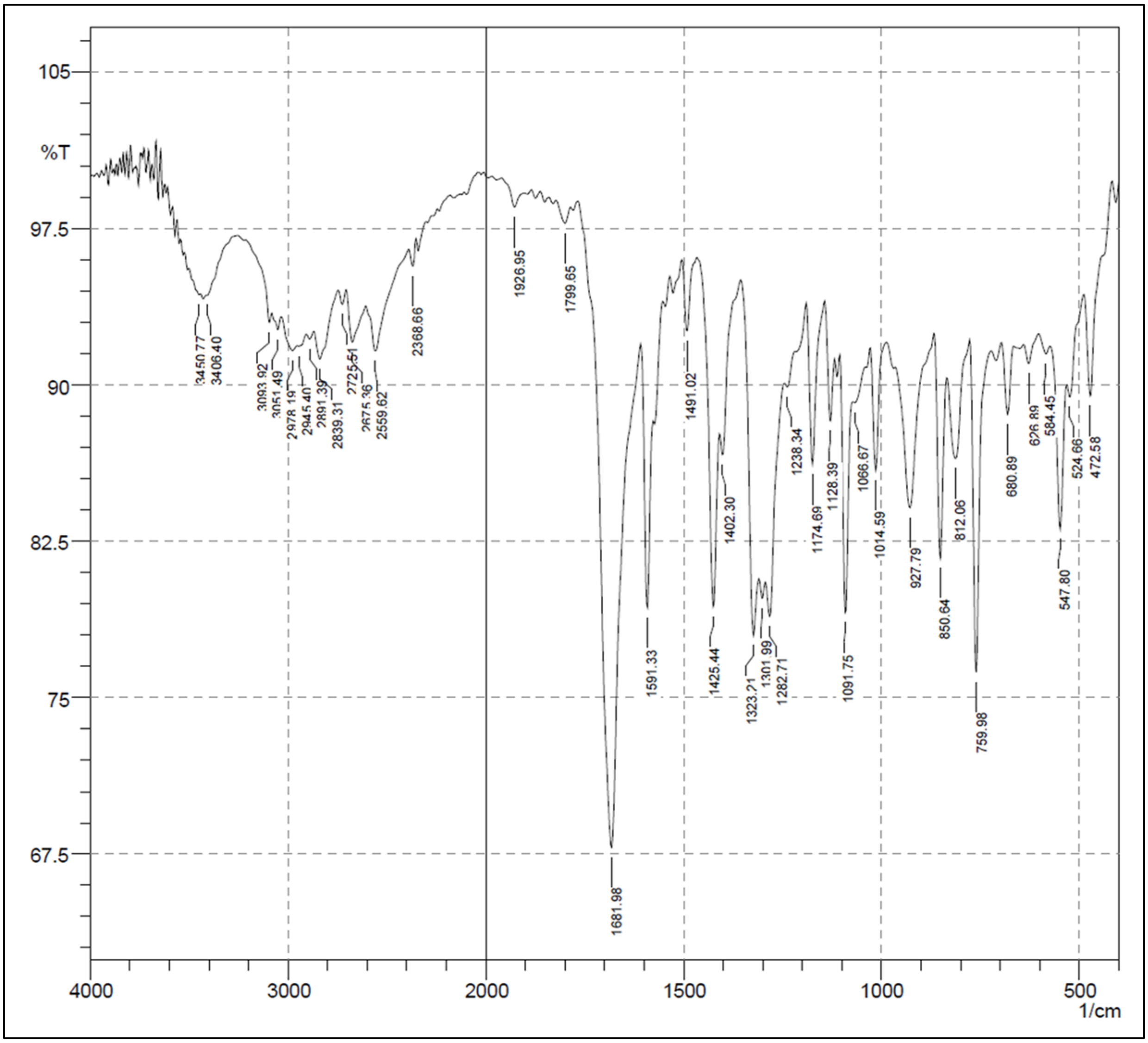

Figure S1. FTIR spectrum of compound 5a

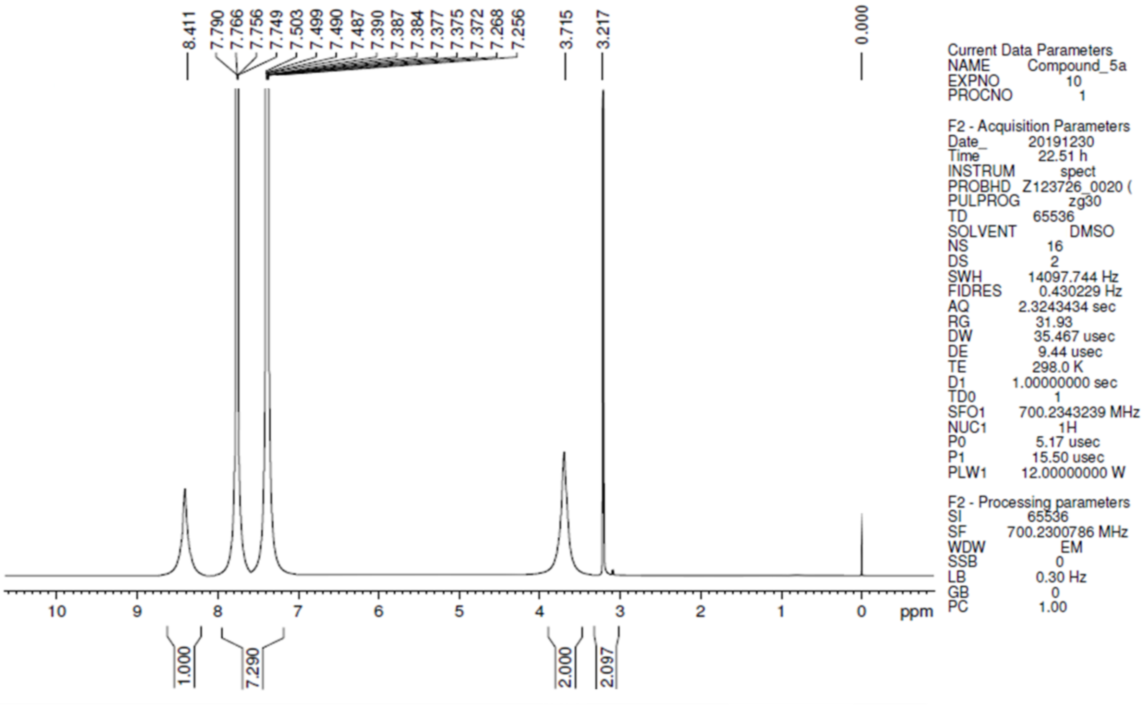

Figure S2. ^{1}H-NMR spectrum of compound 5a

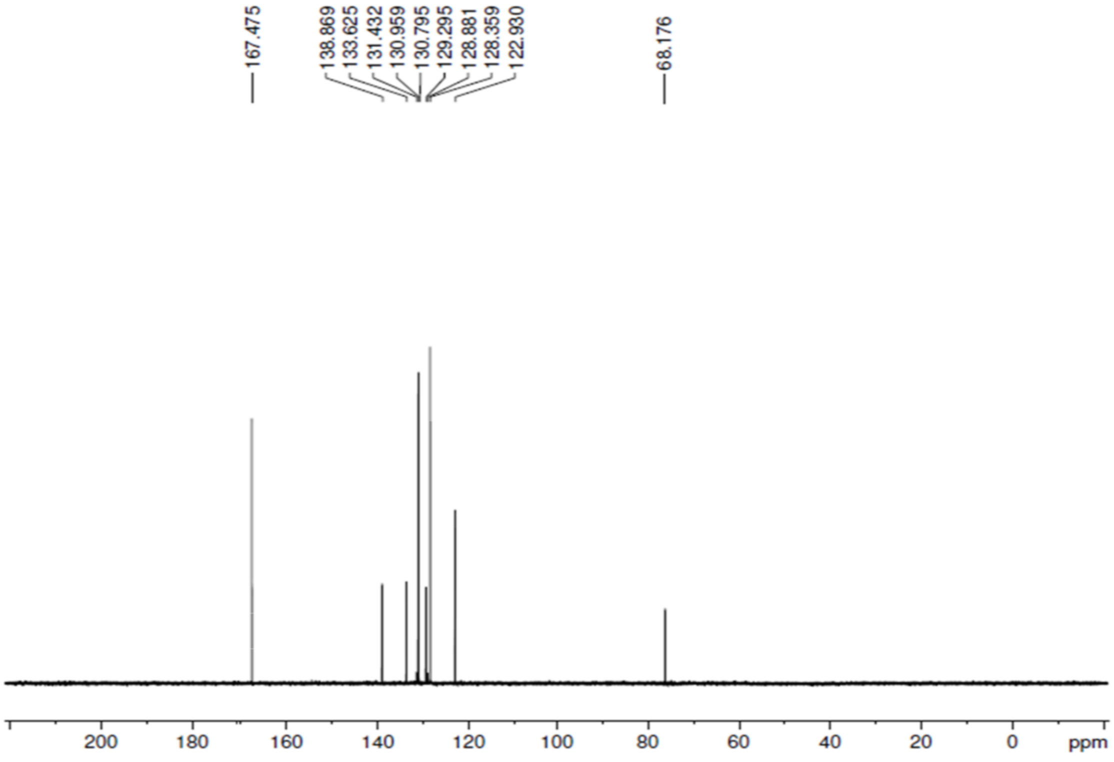

Figure S3. ^{13}C-NMR spectrum of compound 5a

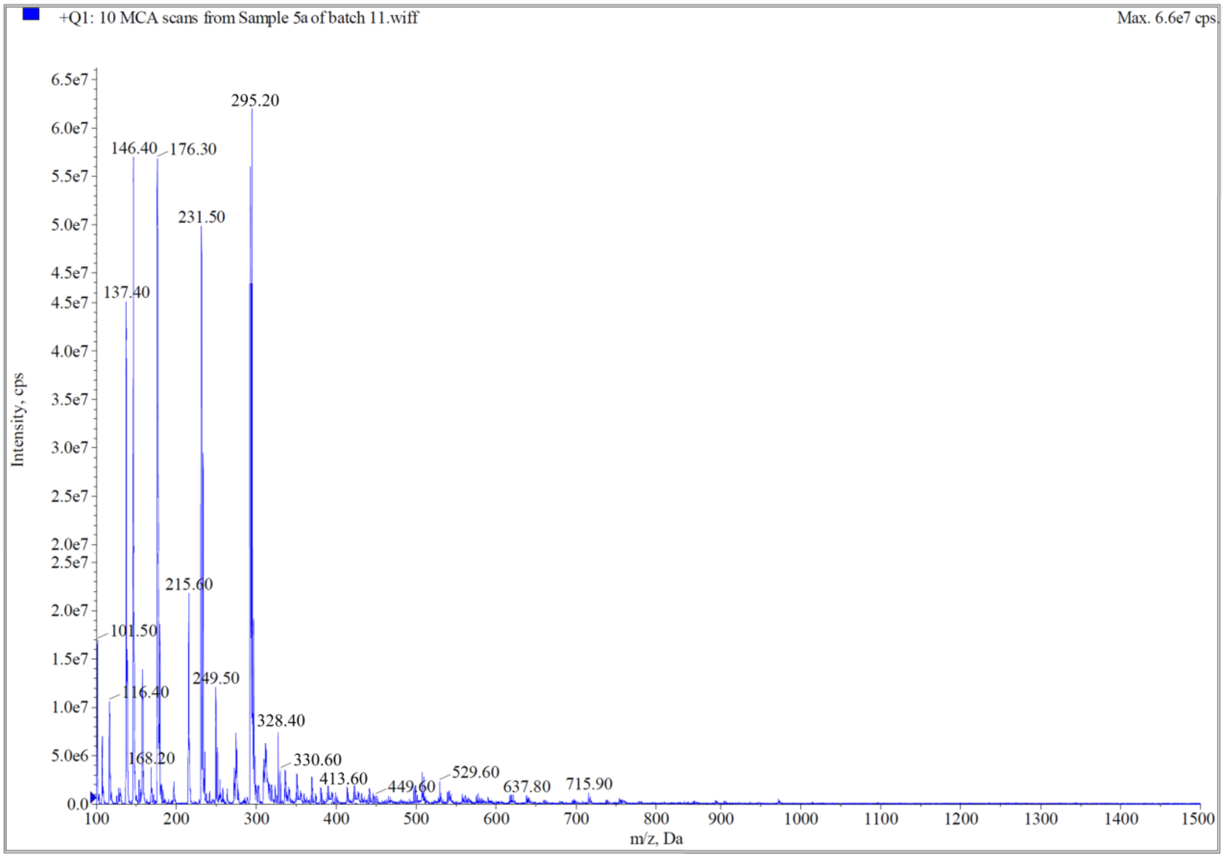

Figure S4. MASS spectrum of compound 5a

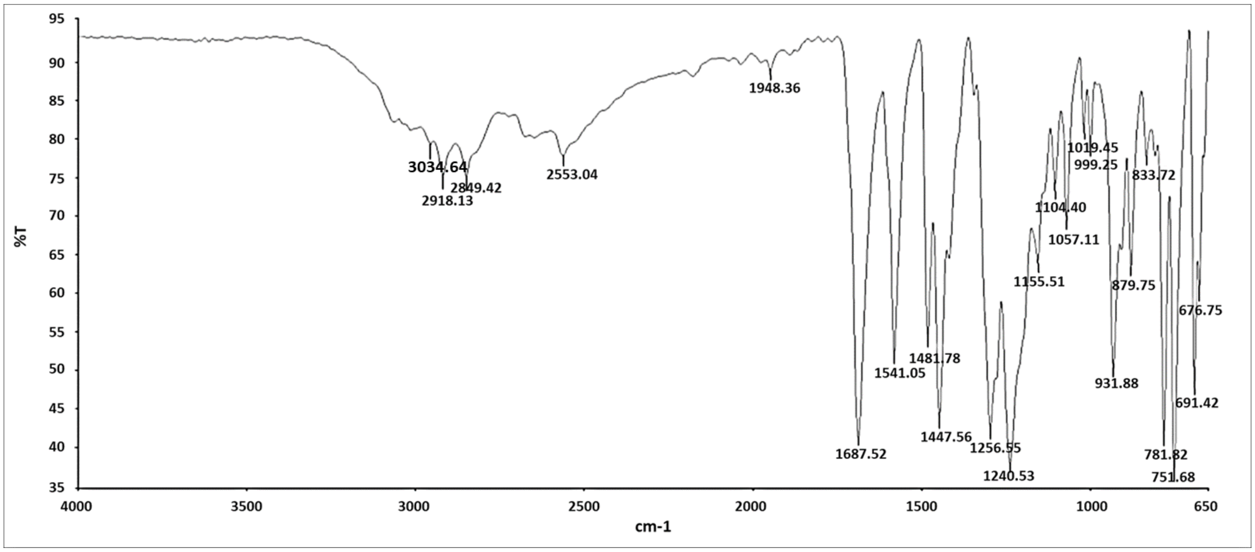

Figure S5. FTIR spectrum of compound 6a

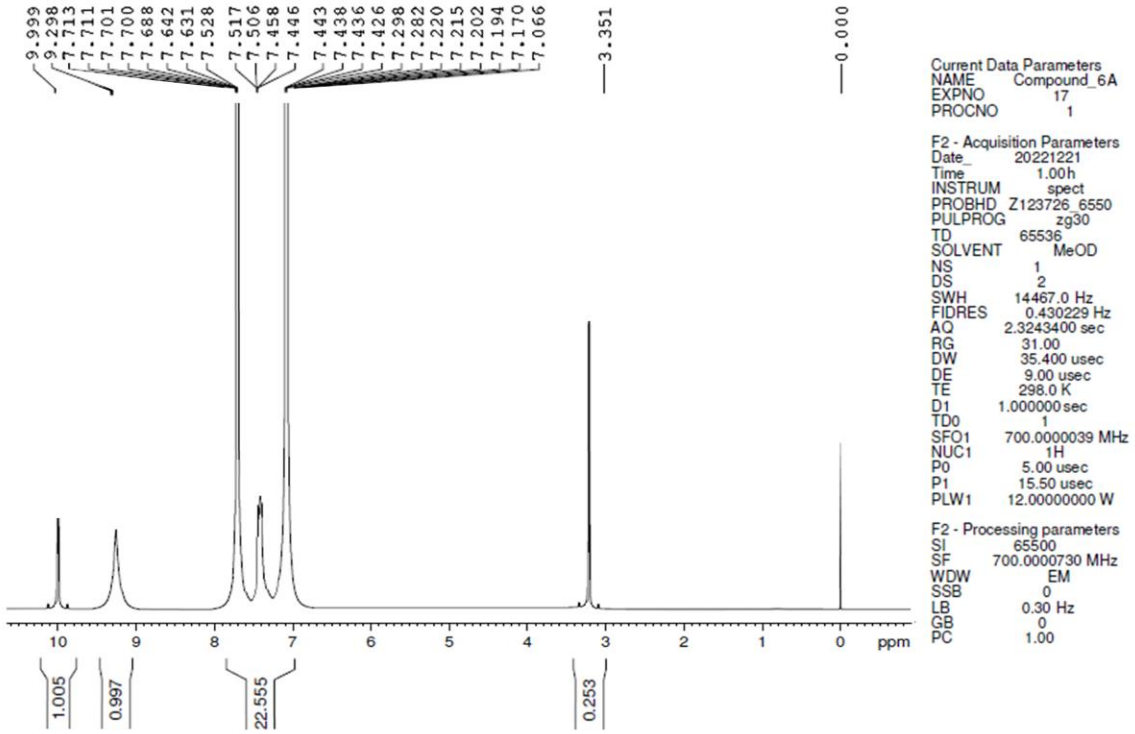

Figure S6. 1H-NMR spectrum of compound 6a

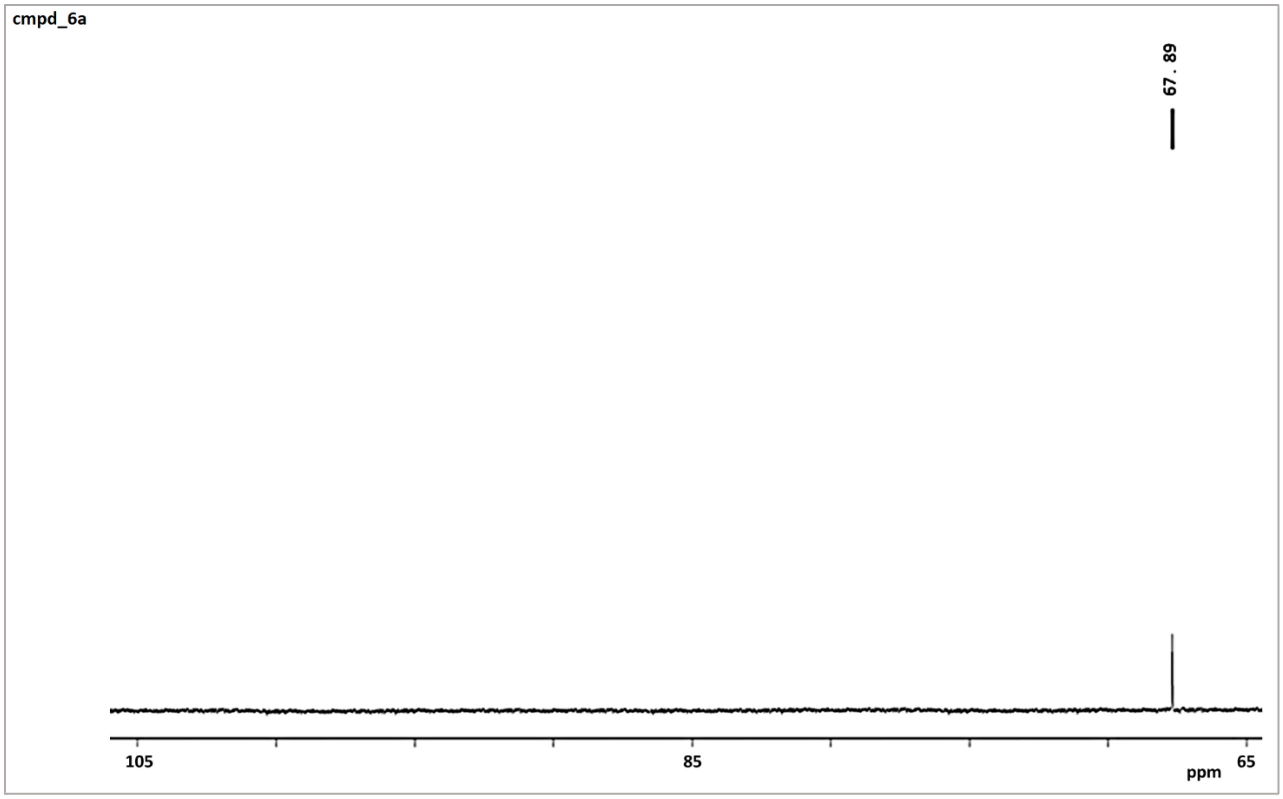

Figure S7a. 13C-NMR spectrum of compound 6a

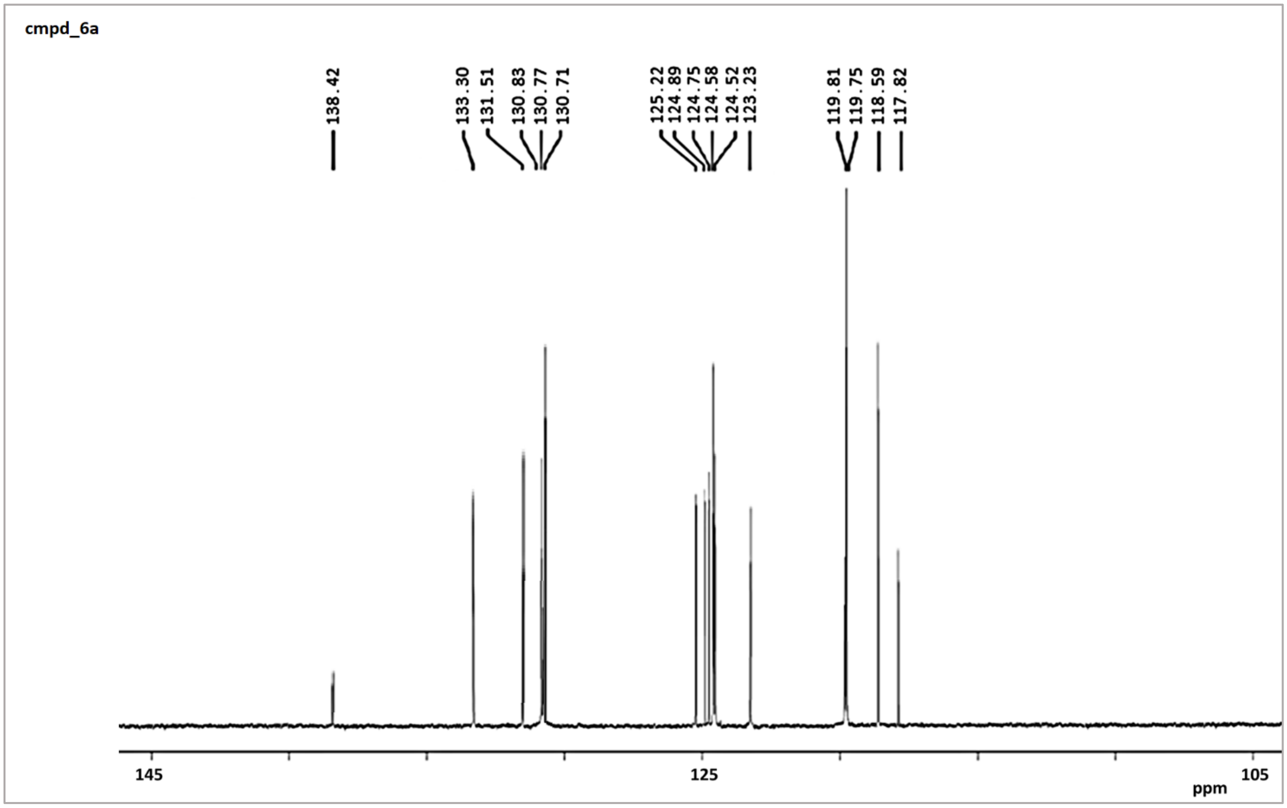

Figure S7b. 13C-NMR spectrum of compound 6a

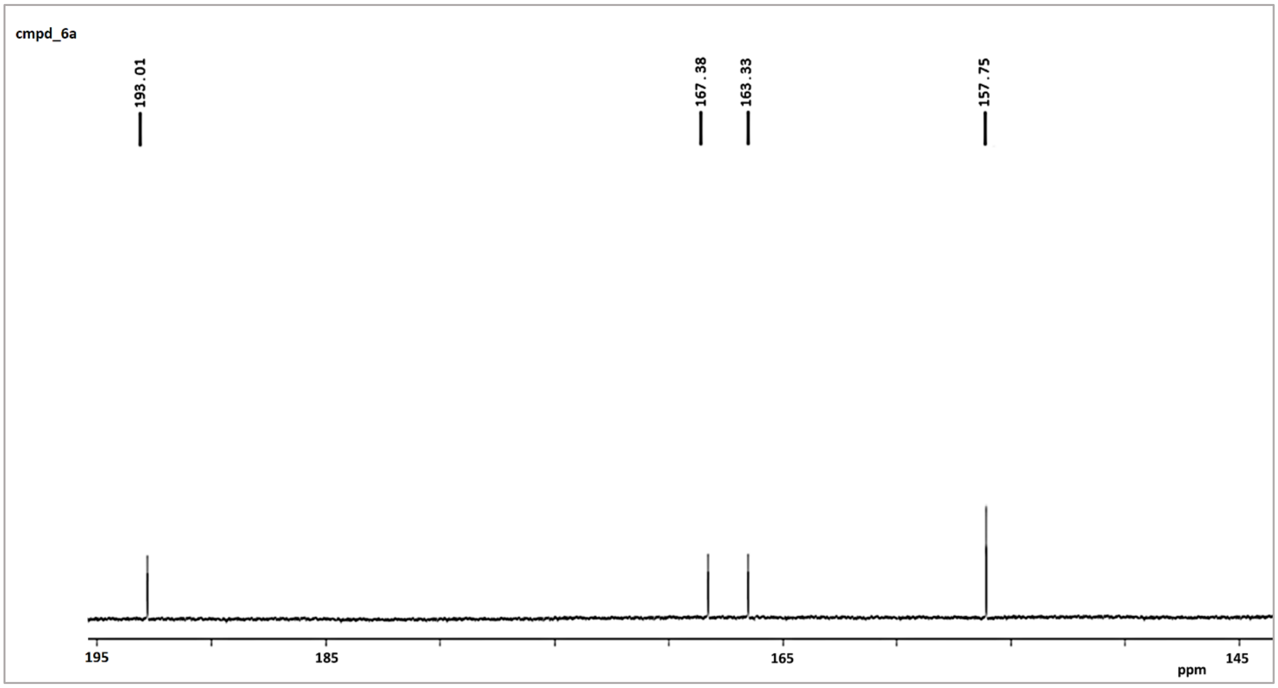

Figure S7c. 13C-NMR spectrum of compound 6a

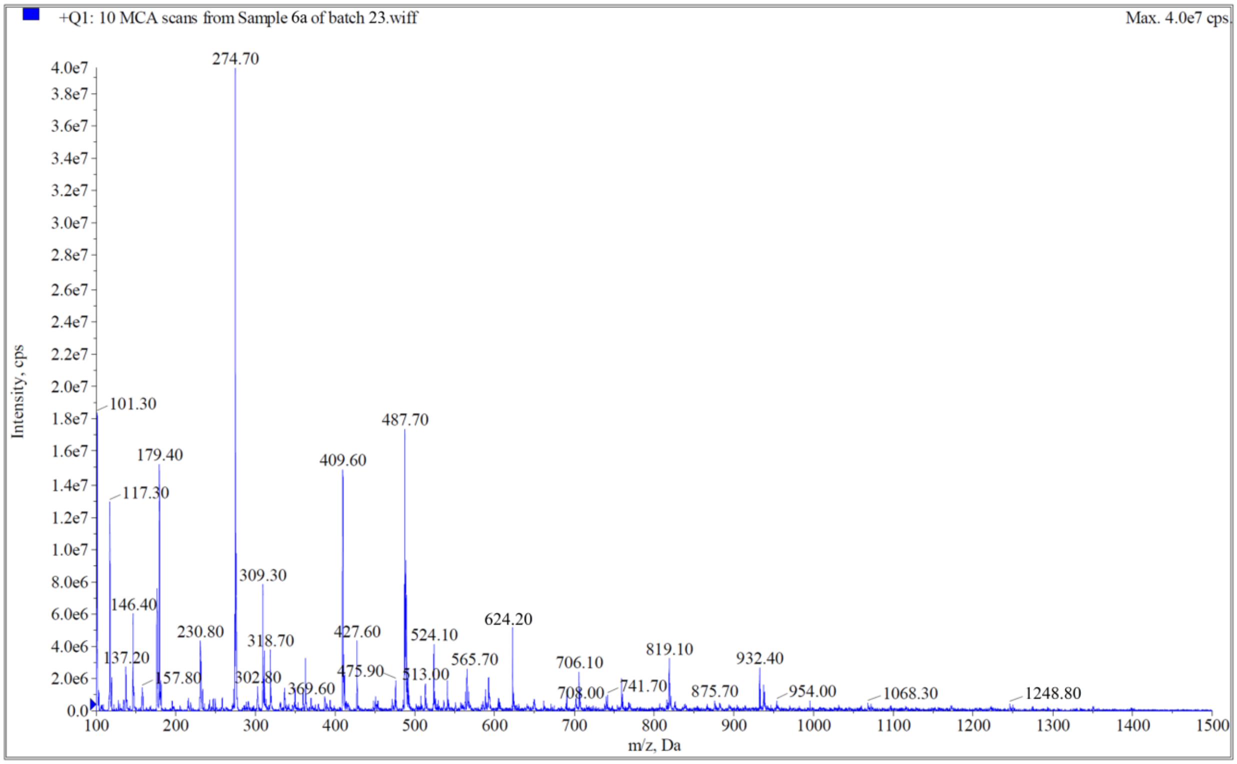

Figure S8. MASS spectrum of compound 6a
